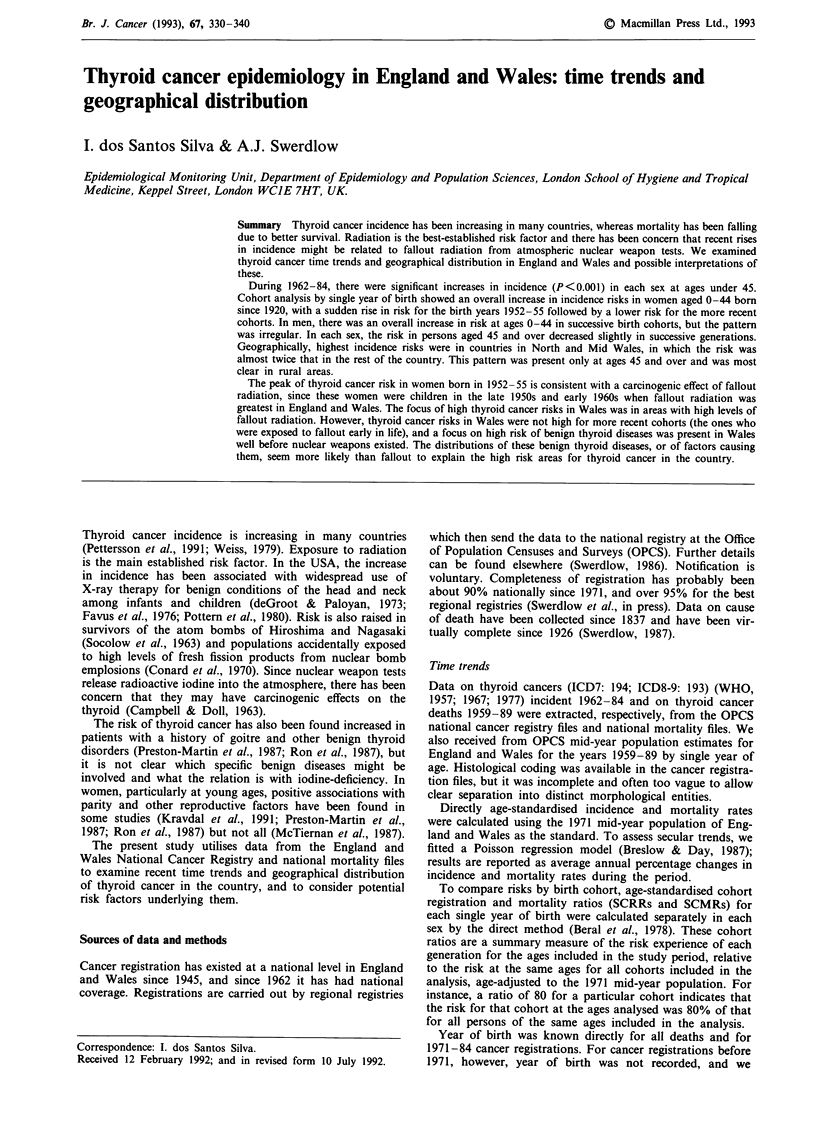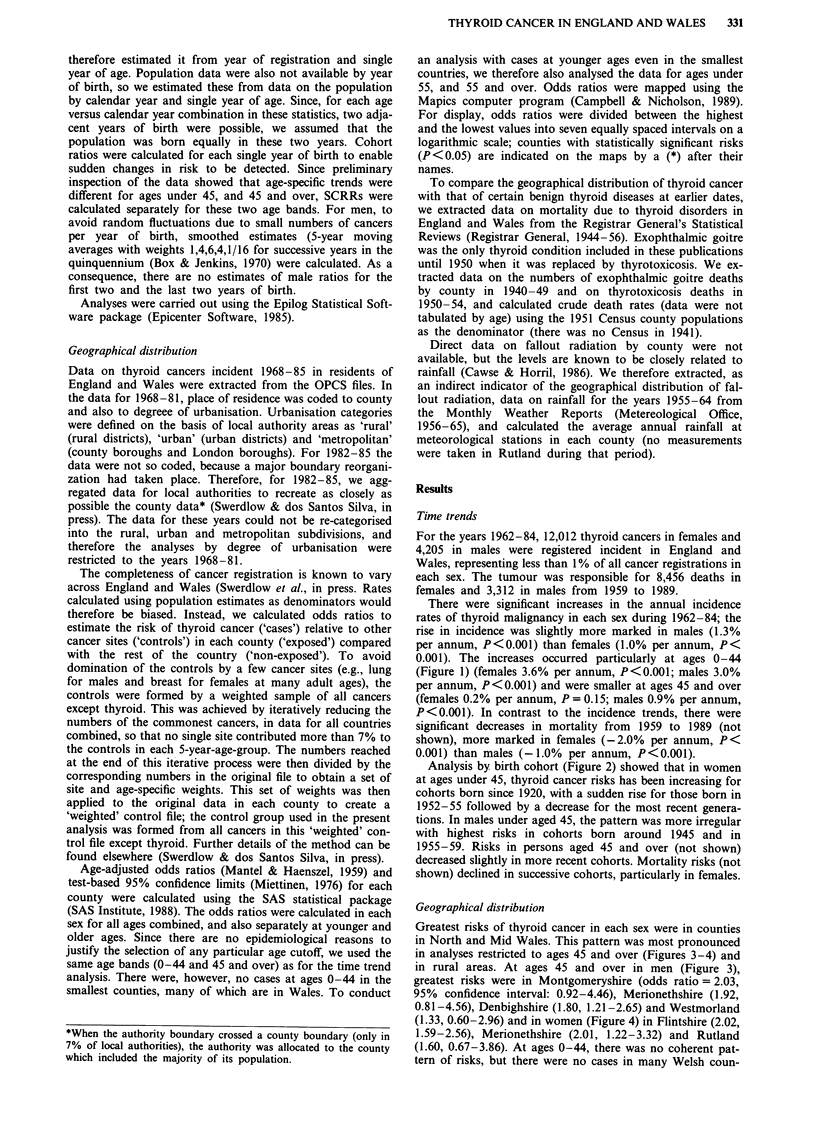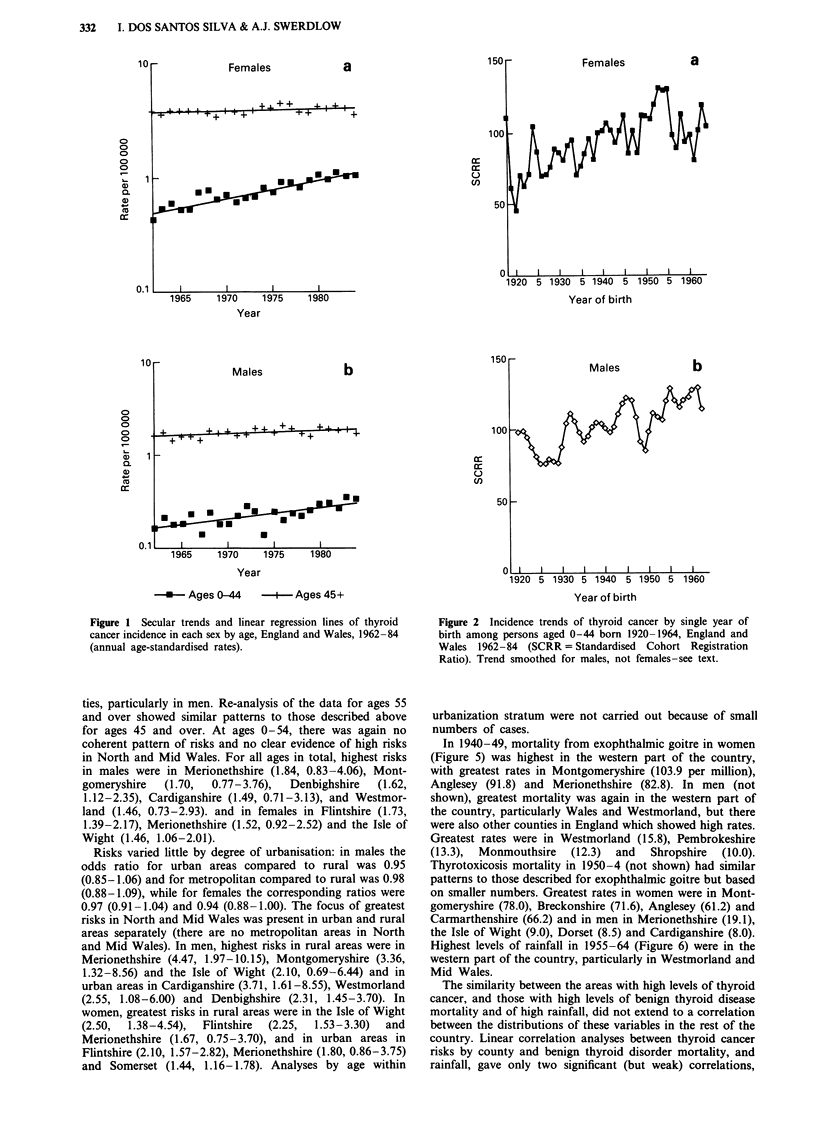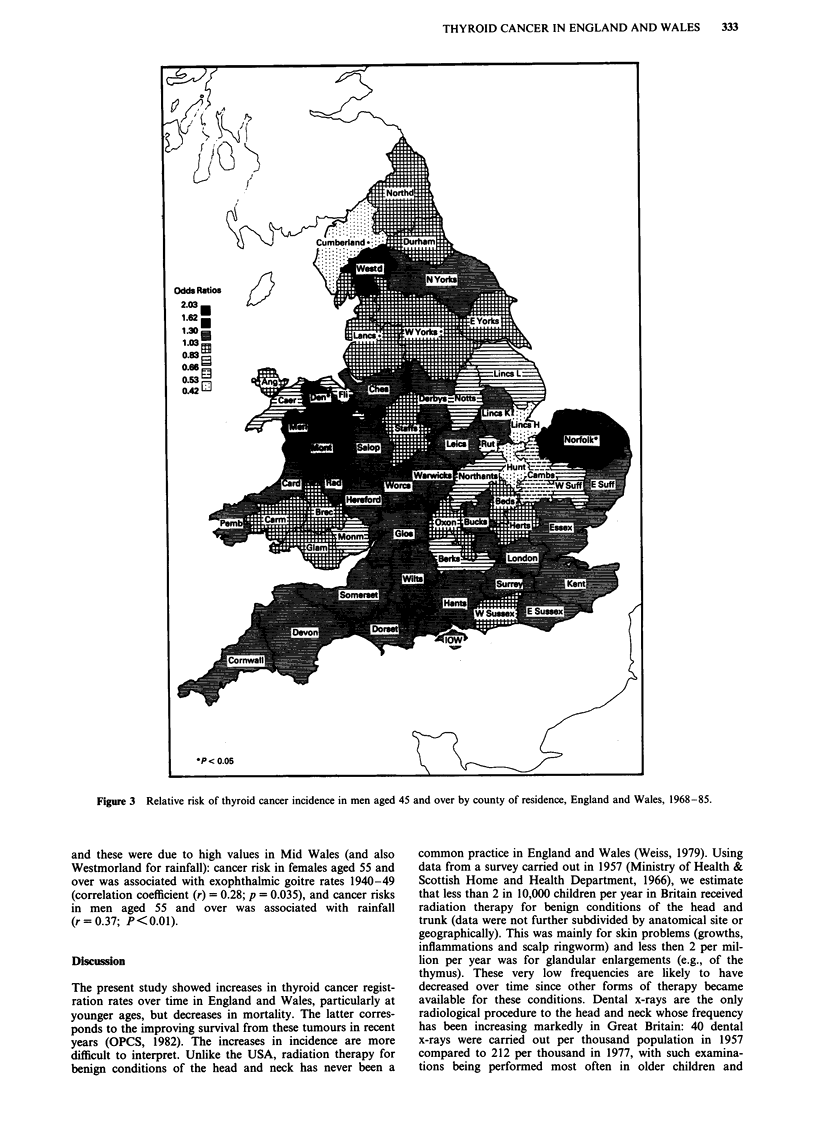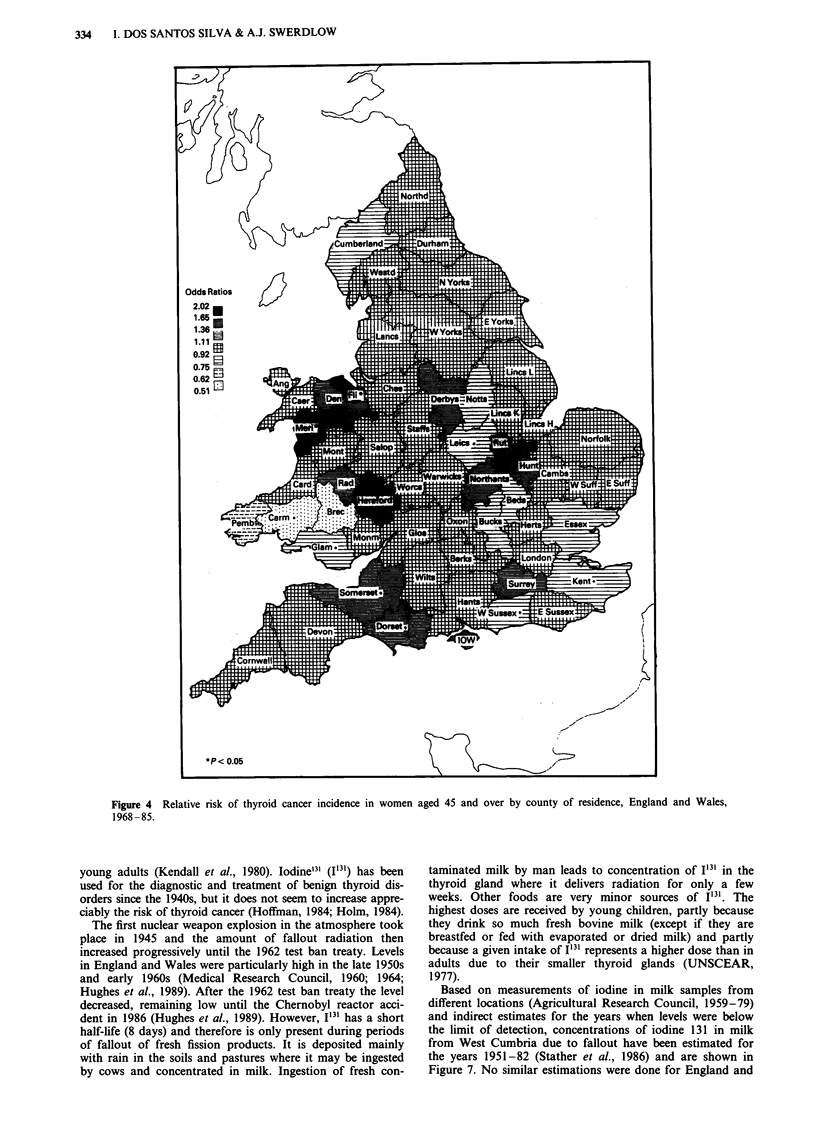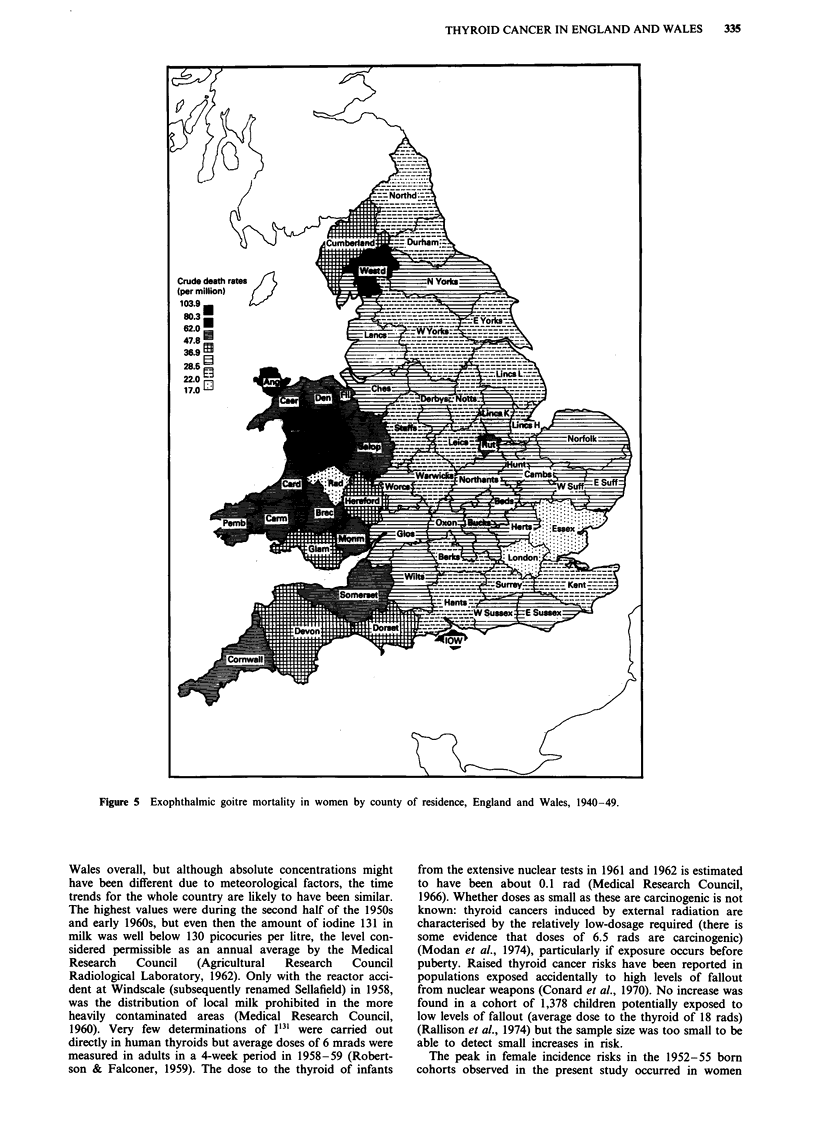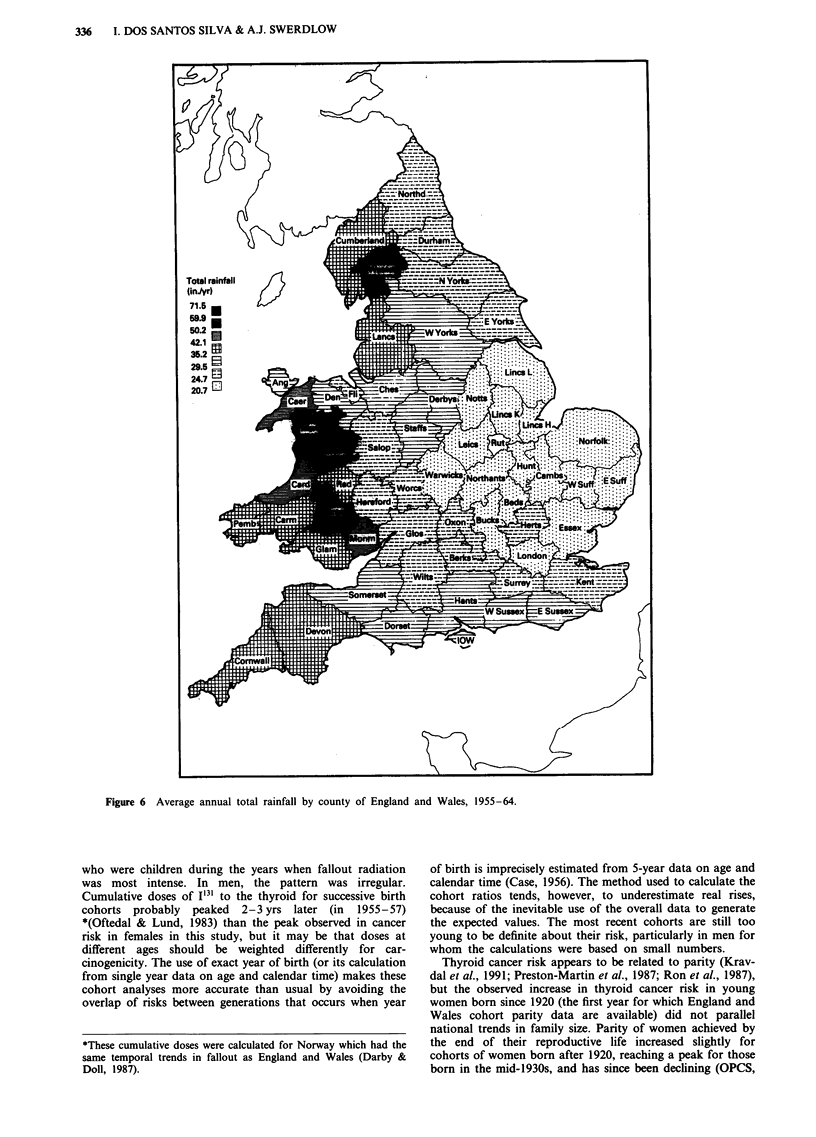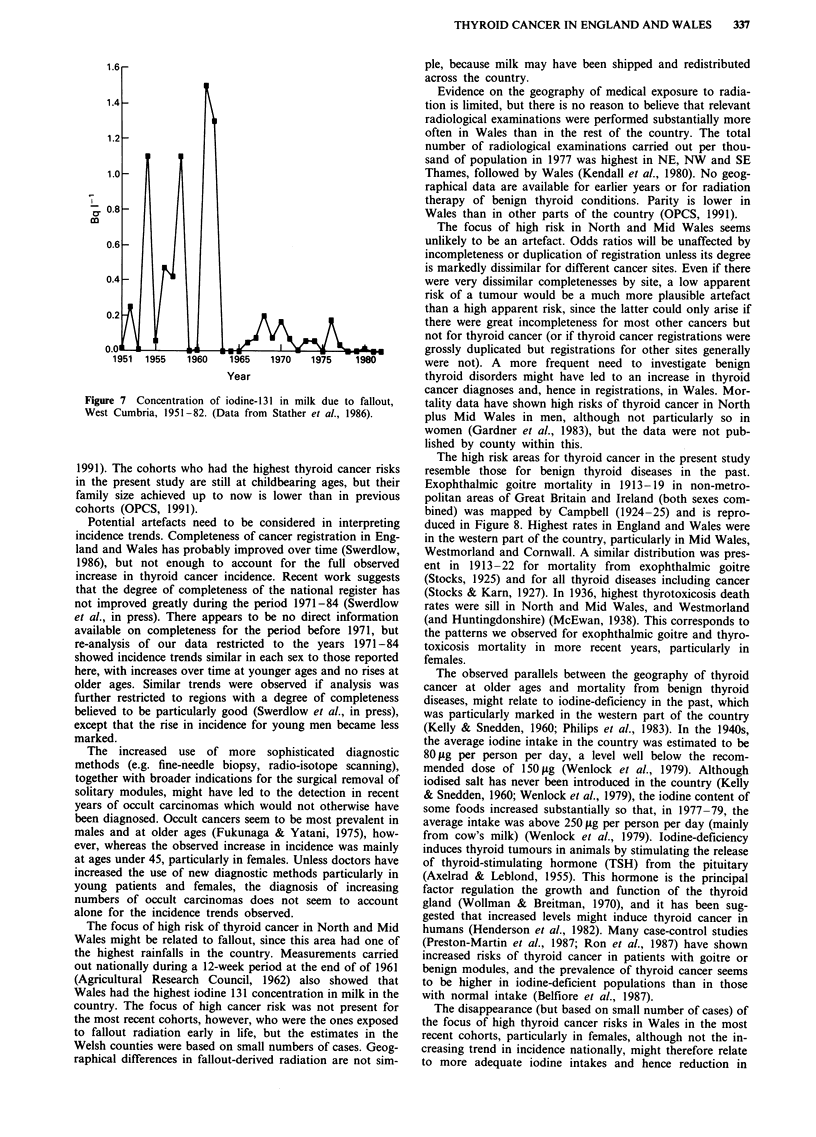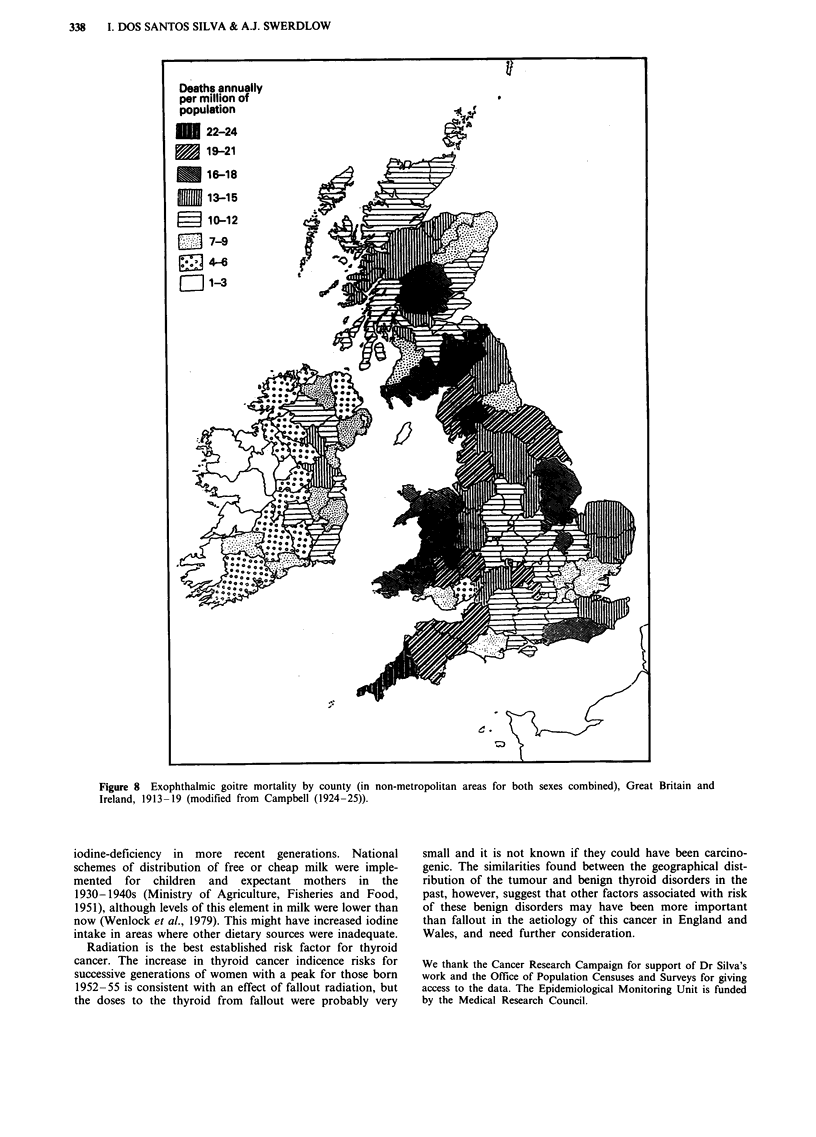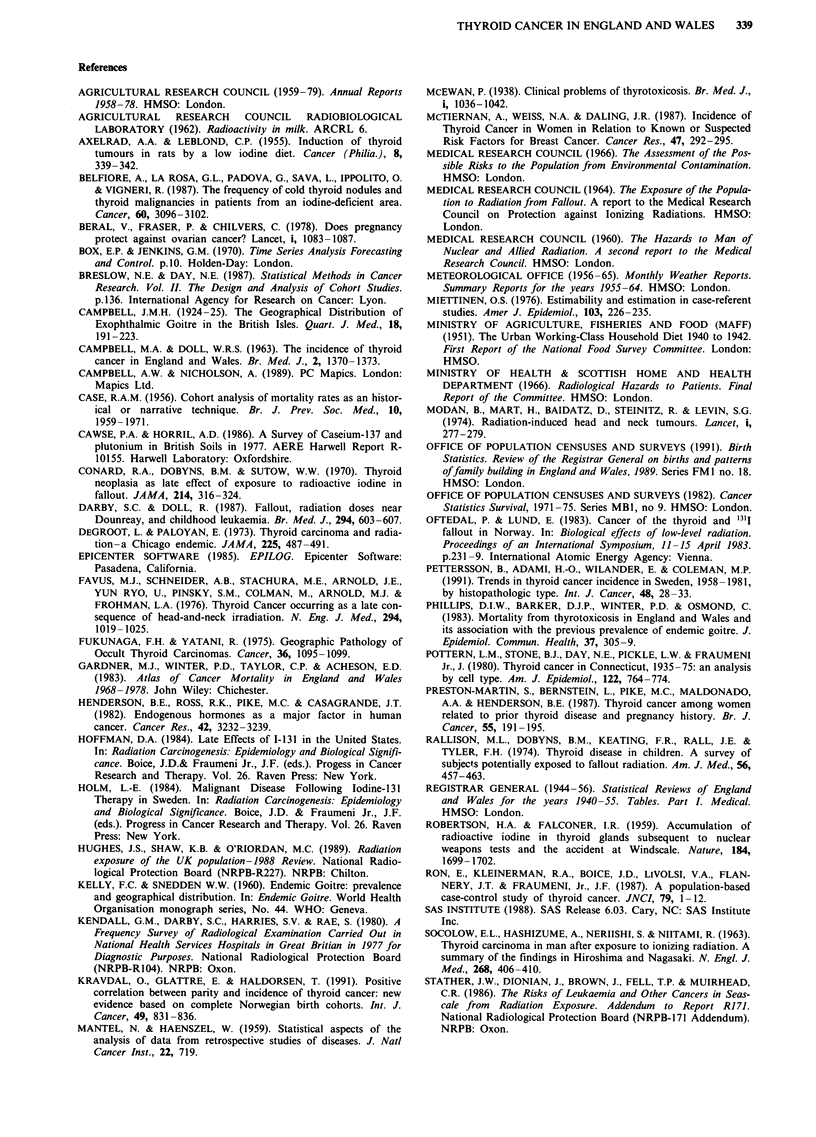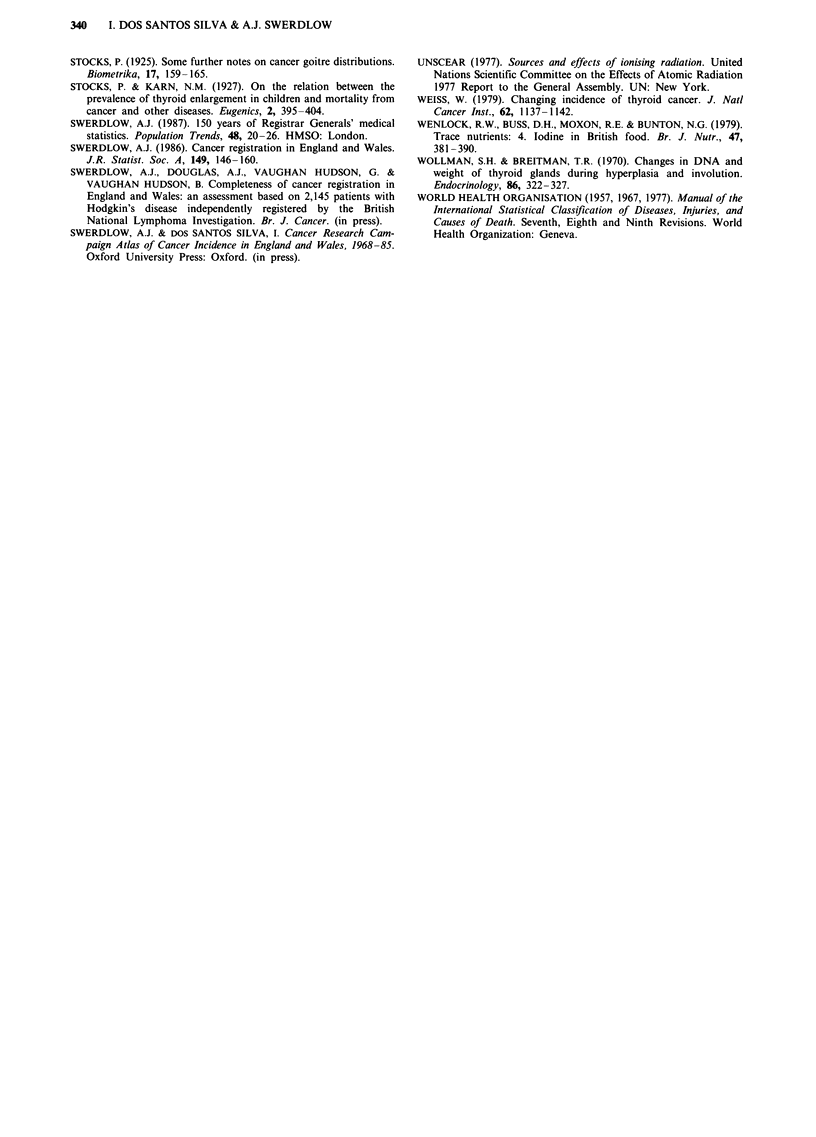# Thyroid cancer epidemiology in England and Wales: time trends and geographical distribution.

**DOI:** 10.1038/bjc.1993.61

**Published:** 1993-02

**Authors:** I. dos Santos Silva, A. J. Swerdlow

**Affiliations:** Department of Epidemiology and Population Sciences, London School of Hygiene and Tropical Medicine, UK.

## Abstract

Thyroid cancer incidence has been increasing in many countries, whereas mortality has been falling due to better survival. Radiation is the best-established risk factor and there has been concern that recent rises in incidence might be related to fallout radiation from atmospheric nuclear weapon tests. We examined thyroid cancer time trends and geographical distribution in England and Wales and possible interpretations of these. During 1962-84, there were significant increases in incidence (P < 0.001) in each sex at ages under 45. Cohort analysis by single year of birth showed an overall increase in incidence risks in women aged 0-44 born since 1920, with a sudden rise in risk for the birth years 1952-55 followed by a lower risk for the more recent cohorts. In men, there was an overall increase in risk at ages 0-44 in successive birth cohorts, but the pattern was irregular. In each sex, the risk in persons aged 45 and over decreased slightly in successive generations. Geographically, highest incidence risks were in countries in North and Mid Wales, in which the risk was almost twice that in the rest of the country. This pattern was present only at ages 45 and over and was most clear in rural areas. The peak of thyroid cancer risk in women born in 1952-55 is consistent with a carcinogenic effect of fallout radiation, since these women were children in the late 1950s and early 1960s when fallout radiation was greatest in England and Wales. The focus of high thyroid cancer risks in Wales was in areas with high levels of fallout radiation. However, thyroid cancer risks in Wales were not high for more recent cohorts (the ones who were exposed to fallout early in life), and a focus on high risk of benign thyroid diseases was present in Wales well before nuclear weapons existed. The distributions of these benign thyroid diseases, or of factors causing them, seem more likely than fallout to explain the high risk areas for thyroid cancer in the country.


Br. J. Cancer (1993), 67, 330–340 © Macmillan Press Ltd., 1993

### Thyroid cancer epidemiology in England and Wales: time trends and geographical distribution

I. dos Santos Silva & A.J. Swerdlow

*Epidemiological Monitoring Unit, Department of Epidemiology and Population Sciences, London School of Hygiene and Tropical Medicine, Keppel Street, London WC1E 7HT, UK.*

**Summary** Thyroid cancer incidence has been increasing in many countries, whereas mortality has been falling due to better survival. Radiation is the best-established risk factor and there has been concern that recent rises in incidence might be related to fallout radiation from atmospheric nuclear weapon tests. We examined thyroid cancer time trends and geographical distribution in England and Wales and possible interpretations of these.

During 1962–84, there were significant increases in incidence ($P<0.001$) in each sex at ages under 45. Cohort analysis by single year of birth showed an overall increase in incidence risks in women aged 0–44 born since 1920, with a sudden rise in risk for the birth years 1952–55 followed by a lower risk for the more recent cohorts. In men, there was an overall increase in risk at ages 0–44 in successive birth cohorts, but the pattern was irregular. In each sex, the risk in persons aged 45 and over decreased slightly in successive generations. Geographically, highest incidence risks were in countries in North and Mid Wales, in which the risk was almost twice that in the rest of the country. This pattern was present only at ages 45 and over and was most clear in rural areas.

The peak of thyroid cancer risk in women born in 1952–55 is consistent with a carcinogenic effect of fallout radiation, since these women were children in the late 1950s and early 1960s when fallout radiation was greatest in England and Wales. The focus of high thyroid cancer risks in Wales was in areas with high levels of fallout radiation. However, thyroid cancer risks in Wales were not high for more recent cohorts (the ones who were exposed to fallout early in life), and a focus on high risk of benign thyroid diseases was present in Wales well before nuclear weapons existed. The distributions of these benign thyroid diseases, or of factors causing them, seem more likely than fallout to explain the high risk areas for thyroid cancer in the country.

Thyroid cancer incidence is increasing in many countries (Pettersson *et al.*, 1991; Weiss, 1979). Exposure to radiation is the main established risk factor. In the USA, the increase in incidence has been associated with widespread use of X-ray therapy for benign conditions of the head and neck among infants and children (deGroot & Paloyan, 1973; Favus *et al.*, 1976; Pottern *et al.*, 1980). Risk is also raised in survivors of the atom bombs of Hiroshima and Nagasaki (Socolow *et al.*, 1963) and populations accidentally exposed to high levels of fresh fission products from nuclear bomb emplosions (Conard *et al.*, 1970). Since nuclear weapon tests release radioactive iodine into the atmosphere, there has been concern that they may have carcinogenic effects on the thyroid (Campbell & Doll, 1963).

The risk of thyroid cancer has also been found increased in patients with a history of goitre and other benign thyroid disorders (Preston-Martin *et al.*, 1987; Ron *et al.*, 1987), but it is not clear which specific benign diseases might be involved and what the relation is with iodine-deficiency. In women, particularly at young ages, positive associations with parity and other reproductive factors have been found in some studies (Kravdal *et al.*, 1991; Preston-Martin *et al.*, 1987; Ron *et al.*, 1987) but not all (McTiernan *et al.*, 1987).

The present study utilises data from the England and Wales National Cancer Registry and national mortality files to examine recent time trends and geographical distribution of thyroid cancer in the country, and to consider potential risk factors underlying them.

### Sources of data and methods

Cancer registration has existed at a national level in England and Wales since 1945, and since 1962 it has had national coverage. Registrations are carried out by regional registries which then send the data to the national registry at the Office of Population Censuses and Surveys (OPCS). Further details can be found elsewhere (Swerdlow, 1986). Notification is voluntary. Completeness of registration has probably been about 90% nationally since 1971, and over 95% for the best regional registries (Swerdlow *et al.*, in press). Data on cause of death have been collected since 1837 and have been virtually complete since 1926 (Swerdlow, 1987).

### *Time trends*

Data on thyroid cancers (ICD7: 194; ICD8-9: 193) (WHO, 1957; 1967; 1977) incident 1962–84 and on thyroid cancer deaths 1959–89 were extracted, respectively, from the OPCS national cancer registry files and national mortality files. We also received from OPCS mid-year population estimates for England and Wales for the years 1959–89 by single year of age. Histological coding was available in the cancer registration files, but it was incomplete and often too vague to allow clear separation into distinct morphological entities.

Directly age-standardised incidence and mortality rates were calculated using the 1971 mid-year population of England and Wales as the standard. To assess secular trends, we fitted a Poisson regression model (Breslow & Day, 1987); results are reported as average annual percentage changes in incidence and mortality rates during the period.

To compare risks by birth cohort, age-standardised cohort registration and mortality ratios (SCRRs and SCMRs) for each single year of birth were calculated separately in each sex by the direct method (Beral *et al.*, 1978). These cohort ratios are a summary measure of the risk experience of each generation for the ages included in the study period, relative to the risk at the same ages for all cohorts included in the analysis, age-adjusted to the 1971 mid-year population. For instance, a ratio of 80 for a particular cohort indicates that the risk for that cohort at the ages analysed was 80% of that for all persons of the same ages included in the analysis.

Year of birth was known directly for all deaths and for 1971–84 cancer registrations. For cancer registrations before 1971, however, year of birth was not recorded, and we

Correspondence: I. dos Santos Silva.
Received 12 February 1992; and in revised form 10 July 1992.

therefore estimated it from year of registration and single year of age. Population data were also not available by year of birth, so we estimated these from data on the population by calendar year and single year of age. Since, for each age versus calendar year combination in these statistics, two adjacent years of birth were possible, we assumed that the population was born equally in these two years. Cohort ratios were calculated for each single year of birth to enable sudden changes in risk to be detected. Since preliminary inspection of the data showed that age-specific trends were different for ages under 45, and 45 and over, SCRRs were calculated separately for these two age bands. For men, to avoid random fluctuations due to small numbers of cancers per year of birth, smoothed estimates (5-year moving averages with weights 1,4,6,4,1/16 for successive years in the quinquennium (Box & Jenkins, 1970) were calculated. As a consequence, there are no estimates of male ratios for the first two and the last two years of birth.

Analyses were carried out using the Epilog Statistical Software package (Epicenter Software, 1985).

*Geographical distribution*

Data on thyroid cancers incident 1968–85 in residents of England and Wales were extracted from the OPCS files. In the data for 1968–81, place of residence was coded to county and also to degreee of urbanisation. Urbanisation categories were defined on the basis of local authority areas as 'rural' (rural districts), 'urban' (urban districts) and 'metropolitan' (county boroughs and London boroughs). For 1982–85 the data were not so coded, because a major boundary reorganization had taken place. Therefore, for 1982–85, we aggregated data for local authorities to recreate as closely as possible the county data* (Swerdlow & dos Santos Silva, in press). The data for these years could not be re-categorised into the rural, urban and metropolitan subdivisions, and therefore the analyses by degree of urbanisation were restricted to the years 1968–81.

The completeness of cancer registration is known to vary across England and Wales (Swerdlow *et al.*, in press. Rates calculated using population estimates as denominators would therefore be biased. Instead, we calculated odds ratios to estimate the risk of thyroid cancer ('cases') relative to other cancer sites ('controls') in each county ('exposed') compared with the rest of the country ('non-exposed'). To avoid domination of the controls by a few cancer sites (e.g., lung for males and breast for females at many adult ages), the controls were formed by a weighted sample of all cancers except thyroid. This was achieved by iteratively reducing the numbers of the commonest cancers, in data for all countries combined, so that no single site contributed more than 7% to the controls in each 5-year-age-group. The numbers reached at the end of this iterative process were then divided by the corresponding numbers in the original file to obtain a set of site and age-specific weights. This set of weights was then applied to the original data in each county to create a 'weighted' control file; the control group used in the present analysis was formed from all cancers in this 'weighted' control file except thyroid. Further details of the method can be found elsewhere (Swerdlow & dos Santos Silva, in press).

Age-adjusted odds ratios (Mantel & Haenszel, 1959) and test-based 95% confidence limits (Miettinen, 1976) for each county were calculated using the SAS statistical package (SAS Institute, 1988). The odds ratios were calculated in each sex for all ages combined, and also separately at younger and older ages. Since there are no epidemiological reasons to justify the selection of any particular age cutoff, we used the same age bands (0–44 and 45 and over) as for the time trend analysis. There were, however, no cases at ages 0–44 in the smallest counties, many of which are in Wales. To conduct an analysis with cases at younger ages even in the smallest countries, we therefore also analysed the data for ages under 55, and 55 and over. Odds ratios were mapped using the Mapics computer program (Campbell & Nicholson, 1989). For display, odds ratios were divided between the highest and the lowest values into seven equally spaced intervals on a logarithmic scale; counties with statistically significant risks ($P<0.05$) are indicated on the maps by a (*) after their names.

To compare the geographical distribution of thyroid cancer with that of certain benign thyroid diseases at earlier dates, we extracted data on mortality due to thyroid disorders in England and Wales from the Registrar General's Statistical Reviews (Registrar General, 1944–56). Exophthalmic goitre was the only thyroid condition included in these publications until 1950 when it was replaced by thyrotoxicosis. We extracted data on the numbers of exophthalmic goitre deaths by county in 1940–49 and on thyrotoxicosis deaths in 1950–54, and calculated crude death rates (data were not tabulated by age) using the 1951 Census county populations as the denominator (there was no Census in 1941).

Direct data on fallout radiation by county were not available, but the levels are known to be closely related to rainfall (Cawse & Horril, 1986). We therefore extracted, as an indirect indicator of the geographical distribution of fallout radiation, data on rainfall for the years 1955–64 from the Monthly Weather Reports (Metereological Office, 1956–65), and calculated the average annual rainfall at meteorological stations in each county (no measurements were taken in Rutland during that period).

**Results**

*Time trends*

For the years 1962–84, 12,012 thyroid cancers in females and 4,205 in males were registered incident in England and Wales, representing less than 1% of all cancer registrations in each sex. The tumour was responsible for 8,456 deaths in females and 3,312 in males from 1959 to 1989.

There were significant increases in the annual incidence rates of thyroid malignancy in each sex during 1962–84; the rise in incidence was slightly more marked in males (1.3% per annum, $P<0.001$) than females (1.0% per annum, $P<0.001$). The increases occurred particularly at ages 0–44 (Figure 1) (females 3.6% per annum, $P<0.001$; males 3.0% per annum, $P<0.001$) and were smaller at ages 45 and over (females 0.2% per annum, $P=0.15$; males 0.9% per annum, $P<0.001$). In contrast to the incidence trends, there were significant decreases in mortality from 1959 to 1989 (not shown), more marked in females (−2.0% per annum, $P<0.001$) than males (−1.0% per annum, $P<0.001$).

Analysis by birth cohort (Figure 2) showed that in women at ages under 45, thyroid cancer risks has been increasing for cohorts born since 1920, with a sudden rise for those born in 1952–55 followed by a decrease for the most recent generations. In males under aged 45, the pattern was more irregular with highest risks in cohorts born around 1945 and in 1955–59. Risks in persons aged 45 and over (not shown) decreased slightly in more recent cohorts. Mortality risks (not shown) declined in successive cohorts, particularly in females.

*Geographical distribution*

Greatest risks of thyroid cancer in each sex were in counties in North and Mid Wales. This pattern was most pronounced in analyses restricted to ages 45 and over (Figures 3–4) and in rural areas. At ages 45 and over in men (Figure 3), greatest risks were in Montgomeryshire (odds ratio = 2.03, 95% confidence interval: 0.92–4.46), Merionethshire (1.92, 0.81–4.56), Denbighshire (1.80, 1.21–2.65) and Westmorland (1.33, 0.60–2.96) and in women (Figure 4) in Flintshire (2.02, 1.59–2.56), Merionethshire (2.01, 1.22–3.32) and Rutland (1.60, 0.67–3.86). At ages 0–44, there was no coherent pattern of risks, but there were no cases in many Welsh coun-

*When the authority boundary crossed a county boundary (only in 7% of local authorities), the authority was allocated to the county which included the majority of its population.

**Figure 1** Secular trends and linear regression lines of thyroid cancer incidence in each sex by age, England and Wales, 1962–84 (annual age-standardised rates).

**Figure 2** Incidence trends of thyroid cancer by single year of birth among persons aged 0–44 born 1920–1964, England and Wales 1962–84 (SCRR = Standardised Cohort Registration Ratio). Trend smoothed for males, not females–see text.

ties, particularly in men. Re-analysis of the data for ages 55 and over showed similar patterns to those described above for ages 45 and over. At ages 0–54, there was again no coherent pattern of risks and no clear evidence of high risks in North and Mid Wales. For all ages in total, highest risks in males were in Merionethshire (1.84, 0.83–4.06), Montgomeryshire (1.70, 0.77–3.76), Denbighshire (1.62, 1.12–2.35), Cardiganshire (1.49, 0.71–3.13), and Westmorland (1.46, 0.73–2.93). and in females in Flintshire (1.73, 1.39–2.17), Merionethshire (1.52, 0.92–2.52) and the Isle of Wight (1.46, 1.06–2.01).

Risks varied little by degree of urbanisation: in males the odds ratio for urban areas compared to rural was 0.95 (0.85–1.06) and for metropolitan compared to rural was 0.98 (0.88–1.09), while for females the corresponding ratios were 0.97 (0.91–1.04) and 0.94 (0.88–1.00). The focus of greatest risks in North and Mid Wales was present in urban and rural areas separately (there are no metropolitan areas in North and Mid Wales). In men, highest risks in rural areas were in Merionethshire (4.47, 1.97–10.15), Montgomeryshire (3.36, 1.32–8.56) and the Isle of Wight (2.10, 0.69–6.44) and in urban areas in Cardiganshire (3.71, 1.61–8.55), Westmorland (2.55, 1.08–6.00) and Denbighshire (2.31, 1.45–3.70). In women, greatest risks in rural areas were in the Isle of Wight (2.50, 1.38–4.54), Flintshire (2.25, 1.53–3.30) and Merionethshire (1.67, 0.75–3.70), and in urban areas in Flintshire (2.10, 1.57–2.82), Merionethshire (1.80, 0.86–3.75) and Somerset (1.44, 1.16–1.78). Analyses by age within urbanization stratum were not carried out because of small numbers of cases.

In 1940–49, mortality from exophthalmic goitre in women (Figure 5) was highest in the western part of the country, with greatest rates in Montgomeryshire (103.9 per million), Anglesey (91.8) and Merionethshire (82.8). In men (not shown), greatest mortality was again in the western part of the country, particularly Wales and Westmorland, but there were also other counties in England which showed high rates. Greatest rates were in Westmorland (15.8), Pembrokeshire (13.3), Monmouthsire (12.3) and Shropshire (10.0). Thyrotoxicosis mortality in 1950–4 (not shown) had similar patterns to those described for exophthalmic goitre but based on smaller numbers. Greatest rates in women were in Montgomeryshire (78.0), Breckonshire (71.6), Anglesey (61.2) and Carmarthenshire (66.2) and in men in Merionethshire (19.1), the Isle of Wight (9.0), Dorset (8.5) and Cardiganshire (8.0). Highest levels of rainfall in 1955–64 (Figure 6) were in the western part of the country, particularly in Westmorland and Mid Wales.

The similarity between the areas with high levels of thyroid cancer, and those with high levels of benign thyroid disease mortality and of high rainfall, did not extend to a correlation between the distributions of these variables in the rest of the country. Linear correlation analyses between thyroid cancer risks by county and benign thyroid disorder mortality, and rainfall, gave only two significant (but weak) correlations,

**Figure 3** Relative risk of thyroid cancer incidence in men aged 45 and over by county of residence, England and Wales, 1968–85.

and these were due to high values in Mid Wales (and also Westmorland for rainfall): cancer risk in females aged 55 and over was associated with exophthalmic goitre rates 1940–49 (correlation coefficient ($r$) = 0.28; $p = 0.035$), and cancer risks in men aged 55 and over was associated with rainfall ($r = 0.37$; $P < 0.01$).

### Discussion

The present study showed increases in thyroid cancer registration rates over time in England and Wales, particularly at younger ages, but decreases in mortality. The latter corresponds to the improving survival from these tumours in recent years (OPCS, 1982). The increases in incidence are more difficult to interpret. Unlike the USA, radiation therapy for benign conditions of the head and neck has never been a common practice in England and Wales (Weiss, 1979). Using data from a survey carried out in 1957 (Ministry of Health & Scottish Home and Health Department, 1966), we estimate that less than 2 in 10,000 children per year in Britain received radiation therapy for benign conditions of the head and trunk (data were not further subdivided by anatomical site or geographically). This was mainly for skin problems (growths, inflammations and scalp ringworm) and less then 2 per million per year was for glandular enlargements (e.g., of the thymus). These very low frequencies are likely to have decreased over time since other forms of therapy became available for these conditions. Dental x-rays are the only radiological procedure to the head and neck whose frequency has been increasing markedly in Great Britain: 40 dental x-rays were carried out per thousand population in 1957 compared to 212 per thousand in 1977, with such examinations being performed most often in older children and

**Figure 4** Relative risk of thyroid cancer incidence in women aged 45 and over by county of residence, England and Wales, 1968–85.

young adults (Kendall *et al.*, 1980). Iodine$^{131}$ ($I^{131}$) has been used for the diagnostic and treatment of benign thyroid disorders since the 1940s, but it does not seem to increase appreciably the risk of thyroid cancer (Hoffman, 1984; Holm, 1984).

The first nuclear weapon explosion in the atmosphere took place in 1945 and the amount of fallout radiation then increased progressively until the 1962 test ban treaty. Levels in England and Wales were particularly high in the late 1950s and early 1960s (Medical Research Council, 1960; 1964; Hughes *et al.*, 1989). After the 1962 test ban treaty the level decreased, remaining low until the Chernobyl reactor accident in 1986 (Hughes *et al.*, 1989). However, $I^{131}$ has a short half-life (8 days) and therefore is only present during periods of fallout of fresh fission products. It is deposited mainly with rain in the soils and pastures where it may be ingested by cows and concentrated in milk. Ingestion of fresh contaminated milk by man leads to concentration of $I^{131}$ in the thyroid gland where it delivers radiation for only a few weeks. Other foods are very minor sources of $I^{131}$. The highest doses are received by young children, partly because they drink so much fresh bovine milk (except if they are breastfed or fed with evaporated or dried milk) and partly because a given intake of $I^{131}$ represents a higher dose than in adults due to their smaller thyroid glands (UNSCEAR, 1977).

Based on measurements of iodine in milk samples from different locations (Agricultural Research Council, 1959–79) and indirect estimates for the years when levels were below the limit of detection, concentrations of iodine 131 in milk from West Cumbria due to fallout have been estimated for the years 1951–82 (Stather *et al.*, 1986) and are shown in Figure 7. No similar estimations were done for England and

**Figure 5** Exophthalmic goitre mortality in women by county of residence, England and Wales, 1940–49.

Wales overall, but although absolute concentrations might have been different due to meteorological factors, the time trends for the whole country are likely to have been similar. The highest values were during the second half of the 1950s and early 1960s, but even then the amount of iodine 131 in milk was well below 130 picocuries per litre, the level considered permissible as an annual average by the Medical Research Council (Agricultural Research Council Radiological Laboratory, 1962). Only with the reactor accident at Windscale (subsequently renamed Sellafield) in 1958, was the distribution of local milk prohibited in the more heavily contaminated areas (Medical Research Council, 1960). Very few determinations of $I^{131}$ were carried out directly in human thyroids but average doses of 6 mrads were measured in adults in a 4-week period in 1958–59 (Robertson & Falconer, 1959). The dose to the thyroid of infants from the extensive nuclear tests in 1961 and 1962 is estimated to have been about 0.1 rad (Medical Research Council, 1966). Whether doses as small as these are carcinogenic is not known: thyroid cancers induced by external radiation are characterised by the relatively low-dosage required (there is some evidence that doses of 6.5 rads are carcinogenic) (Modan *et al.*, 1974), particularly if exposure occurs before puberty. Raised thyroid cancer risks have been reported in populations exposed accidentally to high levels of fallout from nuclear weapons (Conard *et al.*, 1970). No increase was found in a cohort of 1,378 children potentially exposed to low levels of fallout (average dose to the thyroid of 18 rads) (Rallison *et al.*, 1974) but the sample size was too small to be able to detect small increases in risk.

The peak in female incidence risks in the 1952–55 born cohorts observed in the present study occurred in women

**Figure 6** Average annual total rainfall by county of England and Wales, 1955–64.

who were children during the years when fallout radiation was most intense. In men, the pattern was irregular. Cumulative doses of $I^{131}$ to the thyroid for successive birth cohorts probably peaked 2–3 yrs later (in 1955–57) *(Oftedal & Lund, 1983) than the peak observed in cancer risk in females in this study, but it may be that doses at different ages should be weighted differently for carcinogenicity. The use of exact year of birth (or its calculation from single year data on age and calendar time) makes these cohort analyses more accurate than usual by avoiding the overlap of risks between generations that occurs when year of birth is imprecisely estimated from 5-year data on age and calendar time (Case, 1956). The method used to calculate the cohort ratios tends, however, to underestimate real rises, because of the inevitable use of the overall data to generate the expected values. The most recent cohorts are still too young to be definite about their risk, particularly in men for whom the calculations were based on small numbers.

Thyroid cancer risk appears to be related to parity (Kravdal *et al.*, 1991; Preston-Martin *et al.*, 1987; Ron *et al.*, 1987), but the observed increase in thyroid cancer risk in young women born since 1920 (the first year for which England and Wales cohort parity data are available) did not parallel national trends in family size. Parity of women achieved by the end of their reproductive life increased slightly for cohorts of women born after 1920, reaching a peak for those born in the mid-1930s, and has since been declining (OPCS,

*These cumulative doses were calculated for Norway which had the same temporal trends in fallout as England and Wales (Darby & Doll, 1987).

**Figure 7** Concentration of iodine-131 in milk due to fallout, West Cumbria, 1951–82. (Data from Stather *et al.*, 1986).

1991). The cohorts who had the highest thyroid cancer risks in the present study are still at childbearing ages, but their family size achieved up to now is lower than in previous cohorts (OPCS, 1991).

Potential artefacts need to be considered in interpreting incidence trends. Completeness of cancer registration in England and Wales has probably improved over time (Swerdlow, 1986), but not enough to account for the full observed increase in thyroid cancer incidence. Recent work suggests that the degree of completeness of the national register has not improved greatly during the period 1971–84 (Swerdlow *et al.*, in press). There appears to be no direct information available on completeness for the period before 1971, but re-analysis of our data restricted to the years 1971–84 showed incidence trends similar in each sex to those reported here, with increases over time at younger ages and no rises at older ages. Similar trends were observed if analysis was further restricted to regions with a degree of completeness believed to be particularly good (Swerdlow *et al.*, in press), except that the rise in incidence for young men became less marked.

The increased use of more sophisticated diagnostic methods (e.g. fine-needle biopsy, radio-isotope scanning), together with broader indications for the surgical removal of solitary modules, might have led to the detection in recent years of occult carcinomas which would not otherwise have been diagnosed. Occult cancers seem to be most prevalent in males and at older ages (Fukunaga & Yatani, 1975), however, whereas the observed increase in incidence was mainly at ages under 45, particularly in females. Unless doctors have increased the use of new diagnostic methods particularly in young patients and females, the diagnosis of increasing numbers of occult carcinomas does not seem to account alone for the incidence trends observed.

The focus of high risk of thyroid cancer in North and Mid Wales might be related to fallout, since this area had one of the highest rainfalls in the country. Measurements carried out nationally during a 12-week period at the end of of 1961 (Agricultural Research Council, 1962) also showed that Wales had the highest iodine 131 concentration in milk in the country. The focus of high cancer risk was not present for the most recent cohorts, however, who were the ones exposed to fallout radiation early in life, but the estimates in the Welsh counties were based on small numbers of cases. Geographical differences in fallout-derived radiation are not simple, because milk may have been shipped and redistributed across the country.

Evidence on the geography of medical exposure to radiation is limited, but there is no reason to believe that relevant radiological examinations were performed substantially more often in Wales than in the rest of the country. The total number of radiological examinations carried out per thousand of population in 1977 was highest in NE, NW and SE Thames, followed by Wales (Kendall *et al.*, 1980). No geographical data are available for earlier years or for radiation therapy of benign thyroid conditions. Parity is lower in Wales than in other parts of the country (OPCS, 1991).

The focus of high risk in North and Mid Wales seems unlikely to be an artefact. Odds ratios will be unaffected by incompleteness or duplication of registration unless its degree is markedly dissimilar for different cancer sites. Even if there were very dissimilar completenesses by site, a low apparent risk of a tumour would be a much more plausible artefact than a high apparent risk, since the latter could only arise if there were great incompleteness for most other cancers but not for thyroid cancer (or if thyroid cancer registrations were grossly duplicated but registrations for other sites generally were not). A more frequent need to investigate benign thyroid disorders might have led to an increase in thyroid cancer diagnoses and, hence in registrations, in Wales. Mortality data have shown high risks of thyroid cancer in North plus Mid Wales in men, although not particularly so in women (Gardner *et al.*, 1983), but the data were not published by county within this.

The high risk areas for thyroid cancer in the present study resemble those for benign thyroid diseases in the past. Exophthalmic goitre mortality in 1913–19 in non-metropolitan areas of Great Britain and Ireland (both sexes combined) was mapped by Campbell (1924–25) and is reproduced in Figure 8. Highest rates in England and Wales were in the western part of the country, particularly in Mid Wales, Westmorland and Cornwall. A similar distribution was present in 1913–22 for mortality from exophthalmic goitre (Stocks, 1925) and for all thyroid diseases including cancer (Stocks & Karn, 1927). In 1936, highest thyrotoxicosis death rates were sill in North and Mid Wales, and Westmorland (and Huntingdonshire) (McEwan, 1938). This corresponds to the patterns we observed for exophthalmic goitre and thyrotoxicosis mortality in more recent years, particularly in females.

The observed parallels between the geography of thyroid cancer at older ages and mortality from benign thyroid diseases, might relate to iodine-deficiency in the past, which was particularly marked in the western part of the country (Kelly & Snedden, 1960; Philips *et al.*, 1983). In the 1940s, the average iodine intake in the country was estimated to be 80 μg per person per day, a level well below the recommended dose of 150 μg (Wenlock *et al.*, 1979). Although iodised salt has never been introduced in the country (Kelly & Snedden, 1960; Wenlock *et al.*, 1979), the iodine content of some foods increased substantially so that, in 1977–79, the average intake was above 250 μg per person per day (mainly from cow's milk) (Wenlock *et al.*, 1979). Iodine-deficiency induces thyroid tumours in animals by stimulating the release of thyroid-stimulating hormone (TSH) from the pituitary (Axelrad & Leblond, 1955). This hormone is the principal factor regulation the growth and function of the thyroid gland (Wollman & Breitman, 1970), and it has been suggested that increased levels might induce thyroid cancer in humans (Henderson *et al.*, 1982). Many case-control studies (Preston-Martin *et al.*, 1987; Ron *et al.*, 1987) have shown increased risks of thyroid cancer in patients with goitre or benign modules, and the prevalence of thyroid cancer seems to be higher in iodine-deficient populations than in those with normal intake (Belfiore *et al.*, 1987).

The disappearance (but based on small number of cases) of the focus of high thyroid cancer risks in Wales in the most recent cohorts, particularly in females, although not the increasing trend in incidence nationally, might therefore relate to more adequate iodine intakes and hence reduction in

**Figure 8** Exophthalmic goitre mortality by county (in non-metropolitan areas for both sexes combined), Great Britain and Ireland, 1913–19 (modified from Campbell (1924–25)).

iodine-deficiency in more recent generations. National schemes of distribution of free or cheap milk were implemented for children and expectant mothers in the 1930–1940s (Ministry of Agriculture, Fisheries and Food, 1951), although levels of this element in milk were lower than now (Wenlock *et al.*, 1979). This might have increased iodine intake in areas where other dietary sources were inadequate.

Radiation is the best established risk factor for thyroid cancer. The increase in thyroid cancer indicence risks for successive generations of women with a peak for those born 1952–55 is consistent with an effect of fallout radiation, but the doses to the thyroid from fallout were probably very small and it is not known if they could have been carcinogenic. The similarities found between the geographical distribution of the tumour and benign thyroid disorders in the past, however, suggest that other factors associated with risk of these benign disorders may have been more important than fallout in the aetiology of this cancer in England and Wales, and need further consideration.

We thank the Cancer Research Campaign for support of Dr Silva's work and the Office of Population Censuses and Surveys for giving access to the data. The Epidemiological Monitoring Unit is funded by the Medical Research Council.

### References

AGRICULTURAL RESEARCH COUNCIL (1959–79). *Annual Reports 1958–78*. HMSO: London.

AGRICULTURAL RESEARCH COUNCIL RADIOBIOLOGICAL LABORATORY (1962). *Radioactivity in milk*. ARCRL 6.

AXELRAD, A.A. & LEBLOND, C.P. (1955). Induction of thyroid tumours in rats by a low iodine diet. *Cancer (Philia.)*, **8**, 339–342.

BELFIORE, A., LA ROSA, G.L., PADOVA, G., SAVA, L., IPPOLITO, O. & VIGNERI, R. (1987). The frequency of cold thyroid nodules and thyroid malignancies in patients from an iodine-deficient area. *Cancer*, **60**, 3096–3102.

BERAL, V., FRASER, P. & CHILVERS, C. (1978). Does pregnancy protect against ovarian cancer? Lancet, **i**, 1083–1087.

BOX, E.P. & JENKINS, G.M. (1970). *Time Series Analysis Forecasting and Control*. p.10. Holden-Day: London.

BRESLOW, N.E. & DAY, N.E. (1987). *Statistical Methods in Cancer Research. Vol. II. The Design and Analysis of Cohort Studies*. p.136. International Agency for Research on Cancer: Lyon.

CAMPBELL, J.M.H. (1924–25). The Geographical Distribution of Exophthalmic Goitre in the British Isles. *Quart. J. Med.*, **18**, 191–223.

CAMPBELL, M.A. & DOLL, W.R.S. (1963). The incidence of thyroid cancer in England and Wales. *Br. Med. J.*, **2**, 1370–1373.

CAMPBELL, A.W. & NICHOLSON, A. (1989). PC Mapics. London: Mapics Ltd.

CASE, R.A.M. (1956). Cohort analysis of mortality rates as an historical or narrative technique. *Br. J. Prev. Soc. Med.*, **10**, 1959–1971.

CAWSE, P.A. & HORRIL, A.D. (1986). A Survey of Caseium-137 and plutonium in British Soils in 1977. AERE Harwell Report R-10155. Harwell Laboratory: Oxfordshire.

CONARD, R.A., DOBYNS, B.M. & SUTOW, W.W. (1970). Thyroid neoplasia as late effect of exposure to radioactive iodine in fallout. *JAMA*, **214**, 316–324.

DARBY, S.C. & DOLL, R. (1987). Fallout, radiation doses near Dounreay, and childhood leukaemia. *Br. Med. J.*, **294**, 603–607.

DEGROOT, L. & PALOYAN, E. (1973). Thyroid carcinoma and radiation–a Chicago endemic. *JAMA*, **225**, 487–491.

EPICENTER SOFTWARE (1985). *EPILOG*. Epicenter Software: Pasadena, California.

FAVUS, M.J., SCHNEIDER, A.B., STACHURA, M.E., ARNOLD, J.E., YUN RYO, U., PINSKY, S.M., COLMAN, M., ARNOLD, M.J. & FROHMAN, L.A. (1976). Thyroid Cancer occurring as a late consequence of head-and-neck irradiation. *N. Eng. J. Med.*, **294**, 1019–1025.

FUKUNAGA, F.H. & YATANI, R. (1975). Geographic Pathology of Occult Thyroid Carcinomas. *Cancer*, **36**, 1095–1099.

GARDNER, M.J., WINTER, P.D., TAYLOR, C.P. & ACHESON, E.D. (1983). *Atlas of Cancer Mortality in England and Wales 1968–1978*. John Wiley: Chichester.

HENDERSON, B.E., ROSS, R.K., PIKE, M.C. & CASAGRANDE, J.T. (1982). Endogenous hormones as a major factor in human cancer. *Cancer Res.*, **42**, 3232–3239.

HOFFMAN, D.A. (1984). Late Effects of I-131 in the United States. In: *Radiation Carcinogenesis: Epidemiology and Biological Significance*. Boice, J.D.& Fraumeni Jr., J.F. (eds.). Progess in Cancer Research and Therapy. Vol. 26. Raven Press: New York.

HOLM, L.-E. (1984). Malignant Disease Following Iodine-131 Therapy in Sweden. In: *Radiation Carcinogenesis: Epidemiology and Biological Significance*. Boice, J.D. & Fraumeni Jr., J.F. (eds.). Progress in Cancer Research and Therapy. Vol. 26. Raven Press: New York.

HUGHES, J.S., SHAW, K.B. & O'RIORDAN, M.C. (1989). *Radiation exposure of the UK population–1988 Review*. National Radiological Protection Board (NRPB-R227). NRPB: Chilton.

KELLY, F.C. & SNEDDEN W.W. (1960). Endemic Goitre: prevalence and geographical distribution. In: *Endemic Goitre*. World Health Organisation monograph series, No. 44. WHO: Geneva.

KENDALL, G.M., DARBY, S.C., HARRIES, S.V. & RAE, S. (1980). *A Frequency Survey of Radiological Examination Carried Out in National Health Services Hospitals in Great Britian in 1977 for Diagnostic Purposes*. National Radiological Protection Board (NRPB-R104). NRPB: Oxon.

KRAVDAL, O., GLATTRE, E. & HALDORSEN, T. (1991). Positive correlation between parity and incidence of thyroid cancer: new evidence based on complete Norwegian birth cohorts. *Int. J. Cancer*, **49**, 831–836.

MANTEL, N. & HAENSZEL, W. (1959). Statistical aspects of the analysis of data from retrospective studies of diseases. *J. Natl Cancer Inst.*, **22**, 719.

MCEWAN, P. (1938). Clinical problems of thyrotoxicosis. *Br. Med. J.*, **i**, 1036–1042.

MCTIERNAN, A., WEISS, N.A. & DALING, J.R. (1987). Incidence of Thyroid Cancer in Women in Relation to Known or Suspected Risk Factors for Breast Cancer. *Cancer Res.*, **47**, 292–295.

MEDICAL RESEARCH COUNCIL (1966). *The Assessment of the Possible Risks to the Population from Environmental Contamination*. HMSO: London.

MEDICAL RESEARCH COUNCIL (1964). *The Exposure of the Population to Radiation from Fallout*. A report to the Medical Research Council on Protection against Ionizing Radiations. HMSO: London.

MEDICAL RESEARCH COUNCIL (1960). *The Hazards to Man of Nuclear and Allied Radiation. A second report to the Medical Research Council*. HMSO: London.

METEOROLOGICAL OFFICE (1956–65). *Monthly Weather Reports. Summary Reports for the years 1955–64*. HMSO: London.

MIETTINEN, O.S. (1976). Estimability and estimation in case-referent studies. *Amer J. Epidemiol.*, **103**, 226–235.

MINISTRY OF AGRICULTURE, FISHERIES AND FOOD (MAFF) (1951). The Urban Working-Class Household Diet 1940 to 1942. *First Report of the National Food Survey Committee*. London: HMSO.

MINISTRY OF HEALTH & SCOTTISH HOME AND HEALTH DEPARTMENT (1966). *Radiological Hazards to Patients. Final Report of the Committee*. HMSO: London.

MODAN, B., MART, H., BAIDATZ, D., STEINITZ, R. & LEVIN, S.G. (1974). Radiation-induced head and neck tumours. *Lancet*, **i**, 277–279.

OFFICE OF POPULATION CENSUSES AND SURVEYS (1991). *Birth Statistics. Review of the Registrar General on births and patterns of family building in England and Wales, 1989*. Series FM1 no. 18. HMSO: London.

OFFICE OF POPULATION CENSUSES AND SURVEYS (1982). *Cancer Statistics Survival*, 1971–75. Series MB1, no 9. HMSO: London.

OFTEDAL, P. & LUND, E. (1983). Cancer of the thyroid and $^{131}$I fallout in Norway. In: *Biological effects of low-level radiation. Proceedings of an International Symposium, 11–15 April 1983*. p.231–9. International Atomic Energy Agency: Vienna.

PETTERSSON, B., ADAMI, H.-O., WILANDER, E. & COLEMAN, M.P. (1991). Trends in thyroid cancer incidence in Sweden, 1958–1981, by histopathologic type. *Int. J. Cancer*, **48**, 28–33.

PHILLIPS, D.I.W., BARKER, D.J.P., WINTER, P.D. & OSMOND, C. (1983). Mortality from thyrotoxicosis in England and Wales and its association with the previous prevalence of endemic goitre. *J. Epidemiol. Commun. Health*, **37**, 305–9.

POTTERN, L.M., STONE, B.J., DAY, N.E., PICKLE, L.W. & FRAUMENI Jr., J. (1980). Thyroid cancer in Connecticut, 1935–75: an analysis by cell type. *Am. J. Epidemiol.*, **122**, 764–774.

PRESTON-MARTIN, S., BERNSTEIN, L., PIKE, M.C., MALDONADO, A.A. & HENDERSON, B.E. (1987). Thyroid cancer among women related to prior thyroid disease and pregnancy history. *Br. J. Cancer*, **55**, 191–195.

RALLISON, M.L., DOBYNS, B.M., KEATING, F.R., RALL, J.E. & TYLER, F.H. (1974). Thyroid disease in children. A survey of subjects potentially exposed to fallout radiation. *Am. J. Med.*, **56**, 457–463.

REGISTRAR GENERAL (1944–56). *Statistical Reviews of England and Wales for the years 1940–55. Tables. Part I. Medical*. HMSO: London.

ROBERTSON, H.A. & FALCONER, I.R. (1959). Accumulation of radioactive iodine in thyroid glands subsequent to nuclear weapons tests and the accident at Windscale. *Nature*, **184**, 1699–1702.

RON, E., KLEINERMAN, R.A., BOICE, J.D., LIVOLSI, V.A., FLANNERY, J.T. & FRAUMENI, Jr., J.F. (1987). A population-based case-control study of thyroid cancer. *JNCI*, **79**, 1–12.

SAS INSTITUTE (1988). SAS Release 6.03. Cary, NC: SAS Institute Inc.

SOCOLOW, E.L., HASHIZUME, A., NERIISHI, S. & NIITAMI, R. (1963). Thyroid carcinoma in man after exposure to ionizing radiation. A summary of the findings in Hiroshima and Nagasaki. *N. Engl. J. Med.*, **268**, 406–410.

STATHER, J.W., DIONIAN, J., BROWN, J., FELL, T.P. & MUIRHEAD, C.R. (1986). *The Risks of Leukaemia and Other Cancers in Seascale from Radiation Exposure. Addendum to Report R171*. National Radiological Protection Board (NRPB-171 Addendum). NRPB: Oxon.

STOCKS, P. (1925). Some further notes on cancer goitre distributions. *Biometrika*, **17**, 159–165.

STOCKS, P. & KARN, N.M. (1927). On the relation between the prevalence of thyroid enlargement in children and mortality from cancer and other diseases. *Eugenics*, **2**, 395–404.

SWERDLOW, A.J. (1987). 150 years of Registrar Generals' medical statistics. *Population Trends*, **48**, 20–26. HMSO: London.

SWERDLOW, A.J. (1986). Cancer registration in England and Wales. *J.R. Statist. Soc. A*, **149**, 146–160.

SWERDLOW, A.J., DOUGLAS, A.J., VAUGHAN HUDSON, G. & VAUGHAN HUDSON, B. Completeness of cancer registration in England and Wales: an assessment based on 2,145 patients with Hodgkin's disease independently registered by the British National Lymphoma Investigation. *Br. J. Cancer*. (in press).

SWERDLOW, A.J. & DOS SANTOS SILVA, I. *Cancer Research Campaign Atlas of Cancer Incidence in England and Wales, 1968–85*. Oxford University Press: Oxford. (in press).

UNSCEAR (1977). *Sources and effects of ionising radiation*. United Nations Scientific Committee on the Effects of Atomic Radiation 1977 Report to the General Assembly. UN: New York.

WEISS, W. (1979). Changing incidence of thyroid cancer. *J. Natl Cancer Inst.*, **62**, 1137–1142.

WENLOCK, R.W., BUSS, D.H., MOXON, R.E. & BUNTON, N.G. (1979). Trace nutrients: 4. Iodine in British food. *Br. J. Nutr.*, **47**, 381–390.

WOLLMAN, S.H. & BREITMAN, T.R. (1970). Changes in DNA and weight of thyroid glands during hyperplasia and involution. *Endocrinology*, **86**, 322–327.

WORLD HEALTH ORGANISATION (1957, 1967, 1977). *Manual of the International Statistical Classification of Diseases, Injuries, and Causes of Death*. Seventh, Eighth and Ninth Revisions. World Health Organization: Geneva.